# A Study on the Effect of Nickel-Plated Graphite Content on the Microstructure and Properties of AlZn/Nickel-Plated Graphite Composite Cold Spray Coatings

**DOI:** 10.3390/ma18020388

**Published:** 2025-01-16

**Authors:** Linggang Zhou, Zecheng Zheng, Qin Wang, Fangfang Wu, Jing Hong, Shengyi Xie, Hongwei Ni, Qiang Feng, Mengxuan Zhou, Mengzhao Li, Guodong Zhang, Chunxu Pan

**Affiliations:** 1 Taizhou Power Supply Company, State Grid Zhejiang Electric Power Co., Ltd., Taizhou 318000, China; 2 Zhejiang Huadian Equipment Testing and Research Institute Co., Ltd., Hangzhou 311121, China; 3 School of Power and Mechanical Engineering, Wuhan University, Wuhan 430072, China; 4 School of Physical and Technology, Wuhan University, Wuhan 430072, China

**Keywords:** cold spraying technology, aluminum busbar, nickel-plated graphite, AlZn coating, annealing treatment, corrosion resistance

## Abstract

Aluminum and its alloys are widely used in the busbar structures of electrolytic aluminum production. However, they are prone to corrosion and wear damage during use, leading to a decline in current-transmission efficiency and potentially causing safety issues. To repair damaged aluminum busbars, this paper explores the feasibility of using cold spraying technology for surface restoration. Using 6063 aluminum alloy as the substrate, AlZn/nickel-plated graphite composite coatings were applied through cold spraying. The effects of different nickel-plated graphite contents on the microstructure, mechanical properties, and corrosion resistance of the coatings were studied. Annealing treatments (200 °C, 300 °C, 400 °C) were further used to improve the coating’s density and performance. The results show that with an increase in the nickel-plated graphite content, the porosity of the coating gradually increases, while the coating’s density and bond strength improve. Additionally, the annealing treatment significantly enhanced the uniformity and hardness of the coating. Moreover, the cold-sprayed coatings exhibited excellent corrosion resistance, especially in the annealed coatings, which showed superior microstructural stability and lower corrosion current density. This study provides a new technological approach for the repair of aluminum busbars and offers an in-depth discussion on the application of cold spraying technology in the surface restoration of aluminum-based composite materials.

## 1. Introduction

Aluminum and its alloys are widely used in various fields due to their excellent properties [1,2,3]. Currently, the industry mainly produces metallic aluminum through electrolysis, using cryolite–alumina in electrolytic cells to conduct redox reactions to obtain molten aluminum [4,5]. The electrolytic cell is the most crucial equipment in primary aluminum production, where the busbar structure plays a role in connecting and transmitting current between cells. To meet the substantial conductivity demands of electrolytic plants, busbars are designed to be relatively heavy, typically welded from cast billets of a certain size made from aluminum with small amounts of silicon, iron, and copper. During the electrolysis process, the working environment of the busbars is complex, and surface damage often occurs due to corrosion and friction. For undamaged busbars, the voltage drop at various points is generally consistent. However, if the busbar is damaged, the resistance increases under a constant current, leading to voltage drop losses, which can result in significant economic losses and even potential safety issues. Therefore, repairing damaged aluminum busbars is crucial.

Repair methods for aluminum busbars include arc welding, gas welding, self-propagating high-temperature synthesis (SHS), thermal spraying, and cold spraying techniques. In electrolytic plants, complex magnetic fields generated by high currents can cause the magnetic deflection of the arc, affecting welding stability. Gas welding is unaffected by magnetic fields but suffers from heat loss, making it suitable only for thin-plate repairs. Self-propagating high-temperature synthesis welding has advantages such as rapid heat release and resistance to magnetic fields, but existing techniques still face issues like incomplete slag–metal separation, high mold-manufacturing costs, and poor wear resistance of the welds, which limit large-scale applications. Thermal spraying is flexible, highly adaptable, efficient, and cost-effective. However, since aluminum is an oxidation-sensitive metal, problems like oxidation, burning, phase changes, residual stresses, and grain growth can occur during thermal spraying, leading to thermal defects and poor adhesion between the coating and the substrate. Cold spraying technology, an emerging surface engineering technique developed from thermal spraying, differs from traditional thermal spraying in that the powder particles do not melt and remain in a solid state during the cold spraying process. Under the drive of compressed gas, the powder particles impact the substrate surface at supersonic speeds, causing intense plastic deformation and adhesion to form a coating, which effectively avoids thermal defects in the coating preparation of aluminum and other oxidation-sensitive metals [6,7]. Xie et al. successfully prepared a TiB_2_/7075Al composite coating on the surface of 7075Al using cold spraying technology [8]. Salih et al. successfully repaired the damaged surface of Al7075 using cold spraying technology [9]. Saiful et al. developed a coating for laser protection through cold spraying [10]. Cold spraying also features high powder deposition rates and strong coating adhesion. Huishu Wu and colleagues [11] successfully prepared aluminum coatings on Q235 steel surfaces using cold spraying technology, with an average coating adhesion strength of 36.1 MPa, significantly higher than the strength of coatings obtained through thermal spraying. This paper considers using cold spraying technology for the surface repair of damaged aluminum busbars, with aluminum-based materials as the primary spraying materials.

Al, Zn, and Ni are common materials for corrosion-resistant coatings. Li Haixiang et al. [12] prepared Zn50Al coatings on stainless steel substrates and measured the corrosion resistance of the coatings in seawater using electrochemical methods. They found that the ZnAl composite coating provided better protection for the steel substrate compared to pure Al and pure Zn coatings, significantly improving the protection lifespan. Huishu Wu et al. [11] used low-pressure cold spraying technology to successfully deposit graphene-coated Al and Zn powders onto low-carbon steel plates, resulting in a Zn-Gr/Al coating with lower corrosion potential and higher corrosion current, enhancing the cathodic protection capability of the AlZn coating. Given that the bonding between cold-sprayed coatings is mechanical, the presence of minor voids and cracks can reduce the coating’s performance. Many researchers [13,14,15,16] have focused on the further post-treatment of coatings, applying additional techniques and methods for surface modification. Numerous studies indicate that the further surface treatment of Al-based cold-sprayed coatings can significantly improve their microstructure and mechanical properties. Siddique et al. [15] prepared aluminum coatings on magnesium alloys and found that after 300 °C vacuum annealing, the coating’s density increased, grain boundaries decreased, coating adhesion became tighter, wear resistance improved significantly, and corrosion resistance increased tenfold compared to the substrate. Rokni et al. [16] investigated various annealing treatments for 7075 Al alloy coatings, analyzing the effects of different aging temperatures on the coating’s microstructure and performance. Nan et al. [17] conducted laser remelting on Al-Si cold-sprayed coatings, discovering that the laser-remelted coatings became denser with the formation of ceramic-phase Al_2_O_3_, enhancing the coating’s microhardness and reducing the surface roughness from 12.2 μm to 5 μm. Notably, annealing treatment can also induce the formation of intermetallic compounds in the coating. Chunjie Huang [18] applied annealing treatment to Ni-Gr/Al cold-sprayed coatings, generating high-performance intermetallic compounds Al_3_Ni and Al_3_Ni_2_, greatly enhancing the overall performance of the coating.

For the application and repair of aluminum busbars, this paper employs cold spraying technology to prepare AlZn/nickel-plated graphite coatings with varying graphite content and investigates the effects of different components on the cold-sprayed coating’s microstructure and performance. Additionally, the AlZn/20 nickel-plated graphite coating is subjected to annealing treatments at 200 °C, 300 °C, and 400 °C to discuss changes in the coating’s microstructure and performance after annealing.

## 2. Materials and Methods

### 2.1. Materials

#### 2.1.1. Matrix Material

Considering the actual engineering application of coatings for repairing damaged aluminum busbars, this experiment used 6063 aluminum alloy, which closely matches the chemical composition of aluminum busbars, as the substrate. The dimensions of the substrate were 100 × 100 × 3 mm, with its main chemical composition detailed in Table 1. Prior to spraying, the substrate required shot blasting to remove impurities and oxide films from the surface, ensuring better adhesion between the coating and the substrate.

#### 2.1.2. Spray Powder

To align with the composition of busbars and meet their conductivity requirements, aluminum-based composite coatings are used for repairing aluminum busbars, ensuring a dense coating structure, good friction wear resistance, and corrosion resistance. AlZn is a common corrosion-resistant metal coating, but its overall hardness is lower, porosity higher, and friction performance poorer due to the presence of Al. Graphite, a common lubricating phase, can effectively reduce friction. Given graphite’s poor deposition characteristics, a nickel coating is applied to graphite to form nickel-plated graphite, enhancing its adhesion properties.

Thus, this study designs AlZn/nickel-plated graphite powders for the repair coating of aluminum busbars. The nickel-plated graphite powders (75 vol.% Ni and 25 vol.% graphite) and Al-Zn alloy powders (85 wt.% Al and 15 wt.% Zn) are spherical, with a particle size of 20 μm, and all sourced from Huayi Alloy Welding Material Ltd., Dalian, China. Table 2 shows the composition ratios of different samples.

### 2.2. Preparation Process of Cold Spray Coating

In this experiment, cold spraying technology was used to prepare AlZn/nickel-plated graphite coatings. The aluminum–zinc-based coatings incorporated nickel-plated graphite powders to investigate the effects of different coating components on their microstructure and properties.

Powder Preparation: An artificial mixing method was used to homogenize the powders for at least 30 min to ensure the even distribution of all components. After preparation, the powders were stored in a dry environment to prevent moisture absorption.

Pre-treatment: Prior to spraying, the substrate was cleaned with degreaser to remove surface oil. Subsequently, the substrate surface was subjected to shot blasting to eliminate impurities and oxide films, enhancing the bonding strength between the substrate and the coating.

Cold Spraying: The low-pressure cold spraying technique was employed in this experiment. All coatings were produced using a cold spraying device model LP-TCY-II (Beijing Tian Cheng Yu New Material Technology Co., Ltd., Beijing, China). The spraying parameters are detailed in Table 3, and the coating thickness for all samples was greater than 1 mm.

### 2.3. Heat Treatment Process

Annealing involves heating a material to a specific temperature, holding it at that temperature for a certain period, and then cooling it at a controlled rate. This process helps achieve more uniform chemical composition and microstructure in the material, refines the grain size, adjusts hardness, and relieves internal stresses and work-hardening effects.

To prevent the oxidation of the cold-sprayed coatings during the annealing process, a vacuum heat-treatment furnace (FZK-15/11, Hefei, China) was used. The cold-sprayed coatings were cut into suitable sizes, and the furnace heating parameters were set accordingly. Given that the melting point of Zn is only 419.53 °C, to avoid the melting of Zn during annealing, the annealing temperature for the AlZn/nickel-plated graphite coatings was selected to be below 400 °C. Three temperature ranges were used: 200 °C, 300 °C, and 400 °C. The coatings were held at each temperature for 4 h, followed by cooling with the furnace.

### 2.4. Microstructure Characterization

#### 2.4.1. Scanning Electron Microscope

The morphology of the coating and the interface between the coating and substrate were observed using a field emission scanning electron microscope (FESEM, MIRA 3 LMH, TESCAN, Brno, Czech Republic). The composition distribution within the alloy coating and at the coating/substrate interface was detected using an Oxford INCA energy-dispersive spectrometer (EDS, MIRA 3 LMH, TESCAN, Brno, Czech Republic) attached to the scanning electron microscope. Prior to testing, the samples were prepared by cutting them into dimensions of 5 × 5 × 10 mm, encapsulating them using cold mounting, exposing the cross-section for easier grinding, and sequentially polishing with sandpapers of grits 200#, 400#, 800#, 1500#, 2000#, and 3000#. Finally, diamond polishing paste was used on a polishing machine until the surface was free of visible scratches.

#### 2.4.2. X-Ray Diffraction

To investigate the effect of annealing treatment on the composition of the coating, X-ray diffraction (XRD) analysis was performed on the surface of the coating. The instrument used was a XPert Pro (PANalytical B.V., Almelo, The Netherlands), with a scanning range of 5° to 90° and a scanning rate of 2°/min.

### 2.5. Macroscopic Performance Characterization

#### 2.5.1. Microhardness

The hardness of the coating was tested using a Vickers hardness tester (HXS-1000A, Shanghai, China). The polished and ground samples were placed on the stage, and ten different points on the coating were selected for testing. After obtaining ten hardness values, the highest and lowest values were discarded, and the average of the remaining values was calculated to represent the average hardness of the coating. The applied load was 1 N, and the dwell time was 10 s.

#### 2.5.2. Surface Roughness

Surface roughness parameters Sa and Sq of the samples annealed at different temperatures were measured using a surface profilometer (NewView9000, ZYGO, Middlefield, CT, USA). Sa represents the average height of the surface profile, while Sq denotes the standard deviation of the height profile. For each sample, five measurements were taken, and the average value was used.

#### 2.5.3. Friction and Wear Test

The wear resistance of the experimentally prepared coatings was studied at room temperature using a ball-on-disk tribometer (MS-T3000, Lanzhou, China). A silicon nitride ball with a diameter of 3 mm was used as the friction counterface. The testing method involved sliding friction with a rotation radius of 3 mm, a load of 1.5 N, a rotational speed of 200 rpm, and a test duration of 30 min. The instrument used was the MS-T3000.

#### 2.5.4. Corrosion Resistance

The electrochemical performance of the coatings was measured using a CS electrochemical workstation (CS310M, Wuhan, China). A 3.5% NaCl solution was used as the corrosive medium. The potentiodynamic polarization curves of both the as-sprayed and annealed aluminum-based composite coatings were recorded. The electrochemical tests were conducted using a three-electrode system: a saturated calomel electrode as the reference electrode, a platinum foil as the counter electrode, and the aluminum-based composite coating with an area of 1 cm^2^ as the working electrode. The scanning range was from −1 to 1 V, and the scanning rate was 1 mV/s.

Due to the bonding between the cold-sprayed coating and the substrate, only the exposed coating surface was tested in the electrochemical sample preparation. Samples were cut into 10 × 10 mm pieces and fixed using cold mounting material, with copper wire used to connect the anode lead clamp.

#### 2.5.5. Residual Stress

In this experiment, residual stress on the surface of annealed coatings was calculated using nanoindentation technology. Nanoindentation is a technique for determining the mechanical properties of materials at the nanoscale. By applying a load to the surface and obtaining a load–displacement curve, various properties of the material can be calculated. The instrument used was the Nano Indenter G200 (KLATencor, Milpitas, CA, USA).

**Figure 1:**
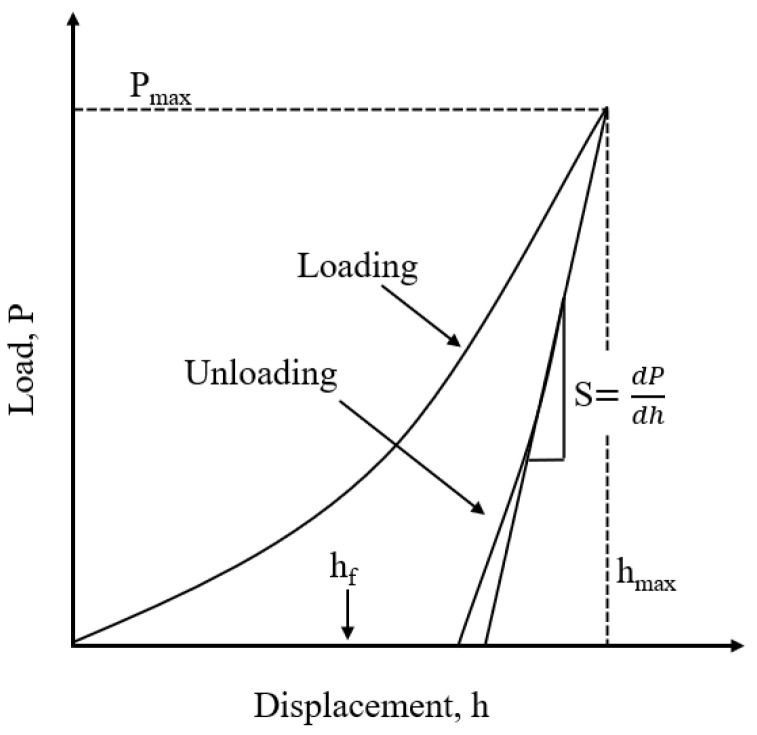
Typical load–displacement curve [19].

**Figure 2:**
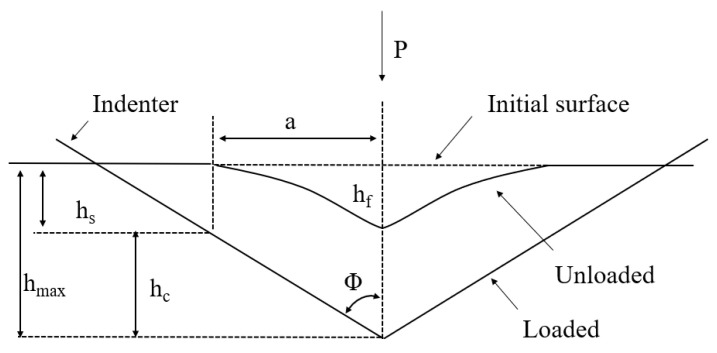
Indentation parameters after unloading [19].

Figure 1 shows a typical load–displacement curve. The left side represents the loading curve, and the right side represents the unloading curve. Here, h_max_ is the maximum displacement, or maximum penetration depth; h_f_ is the plastic depth, which is the rebound displacement due to plastic deformation recovery; and P_max_ is the maximum load. For perfectly elastic materials, there is no plastic deformation, so the material can return to its pre-loading state upon unloading, resulting in overlapping loading and unloading curves. Conversely, for perfectly plastic materials, there is no recovery, and the unloading curve will be vertical relative to the horizontal axis. Figure 2 is a schematic of the indentation after unloading, where h_max_ is the maximum depth of the indenter penetration, h_c_ is the maximum contact depth, h_s_ is the height deviation from the surface contact perimeter, and h_f_ is the plastic depth.

Based on typical load–displacement curves and various parameters, many data regarding the coating surface can be calculated.

(1) Contact Stiffness: According to the characteristics of the load–displacement curve, Oliver–Pharr [19] used a power function for fitting. The Formula (1) is:

(1) $$P=B{\left(h-h_{f}\right)}^{m}$$

where *B* and m are constants obtained via the least squares method, *h_f_* represents the plastic depth directly obtained from the load–displacement curve, and h is the total depth. The material stiffness is then directly obtained by differentiating Equation (1).

(2) Contact Depth: As observed in Figure 2, the maximum indentation depth is equal to the contact depth plus the deviation height from the surface contact perimeter,

(2) $$h=h_{c}+h_{s}$$

According to the Sneddon formula [20], it is known that:

(3) $$h_{s}=\varepsilon \frac{P_{\max}}{S}$$

where *ε* is a parameter related to the shape of the test indenter, with a value of 0.75 for a Berkovich indenter. By substituting Equation (3) into Equation (2), the formula for calculating the contact depth can be directly obtained. For most materials, the contact depth equals the plastic depth, *h_c_* = *h_f_*.

(3) Contact Area: The contact area refers to the effective contact area between the indenter and the material being measured, typically considered as the projected area of the indentation at the contact depth. In this study, an empirical formula is used. For a Berkovich indenter, the empirical formula for the contact area is given by:

(4) $$A\approx 24.5h_{c}^{2}$$

(4) Hardness: Hardness is calculated using the conventional formula:

(5) $$H=\frac{P_{\max}}{A}$$

where *P*_max_ is the maximum load and *A* is the effective contact area. The parameters for the nanoindentation experiment are shown in Table 4.

## 3. Results

### 3.1. Microstructure of AlZn Cold Spray Coating

**Figure 3:**
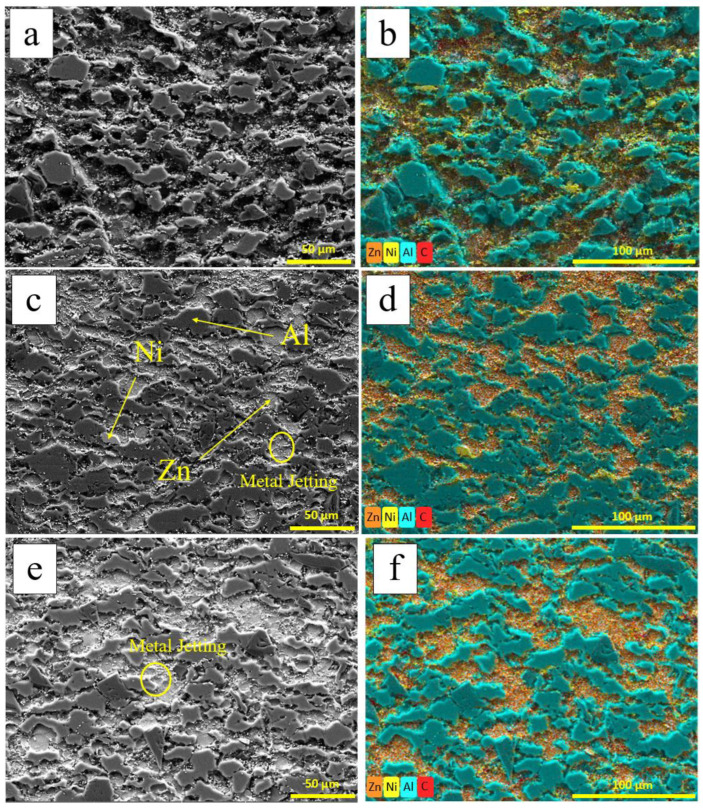
SEM and EDS of AlZn/Gr-Ni coating section: (**a**,**b**) 10Gr-Ni; (**c**,**d**) 20Gr-Ni; (**e**,**f**) 30Gr-Ni.

Graphite is a common reinforcement particle used to enhance the friction performance of coatings. However, graphite is hard and difficult to deform, making deposition challenging during low-pressure cold spraying. Coating graphite with a layer of Ni can effectively improve its lubrication properties and enhance its plastic deformability, aiding in its deposition. Figure 3 shows the microstructure of AlZn composite coatings with varying amounts of nickel-coated graphite: 10%, 20%, and 30%. The gray areas, matching the color of the matrix, represent Al, which constitutes the majority of the coating, while the white regions indicate Zn and some nickel-coated graphite. Nickel-coated graphite should not exceed 50% to ensure the good deposition of the coating.

Figure 3 shows a three-dimensional cross-section after polishing. The black areas in Figure 3a are shadows caused by height differences. This is likely due to the hardness of nickel-coated graphite, which is less deformable compared to the softer, more tightly bonded Al and Zn. The nickel-coated graphite does not bond as well with other particles and is removed during polishing. The gray parts are Al and the white parts are Zn. Due to the flaking of graphite, Ni is concentrated in the recesses of the section and at the AlZn interface. Severe plastic deformation of Al and Zn particles can be observed under the compaction of nickel-coated graphite.

Upon close examination, dispersed white particles can be observed at the AlZn boundary, which may be due to the adiabatic shear instability of the nickel-coated graphite during deposition, with metal jet traces from the nickel on the graphite surface. In cold spraying, the adiabatic shear instability of powder particles during deposition leads to tangential strain, resulting in metal jetting and the extrusion of both the powder and substrate materials, as indicated by the yellow circle in Figure 3.

According to measurements in Photoshop, the porosities of the coatings with different nickel-coated graphite contents are 2.3%, 2.4%, and 2.8%, respectively. The porosity of AlZn/nickel-coated graphite coatings increase with the amount of nickel-coated graphite added. On one hand, the nickel-coated graphite acts as a reinforcing phase, intensifying the deformation of Al and Zn particles and increasing the coating density. On the other hand, the difficulty in deforming graphite and its loose bonding, combined with the increased collisions between particles as their content rises, reduces the effectiveness of the adhesive layer of Al, leading to a decreased coating density. The latter effect dominates, resulting in lower coating density.

### 3.2. The Effect of Annealing Treatment on the Microstructure of Coatings

Under certain conditions, the rate of diffusion is primarily determined by the diffusion coefficient, which is influenced by the diffusion activation energy and temperature. The Formula (6) is:

(6) $$D=D_{0}\exp(-\frac{Q}{RT})$$

where D_0_ is the diffusion constant (m/s^2^), *Q* is the diffusion activation energy (J·mol^−1^), *R* is the Boltzmann constant, and *T* is the thermodynamic temperature (K). According to this formula, the diffusion coefficient of the material increases with rising temperature.

**Figure 4:**
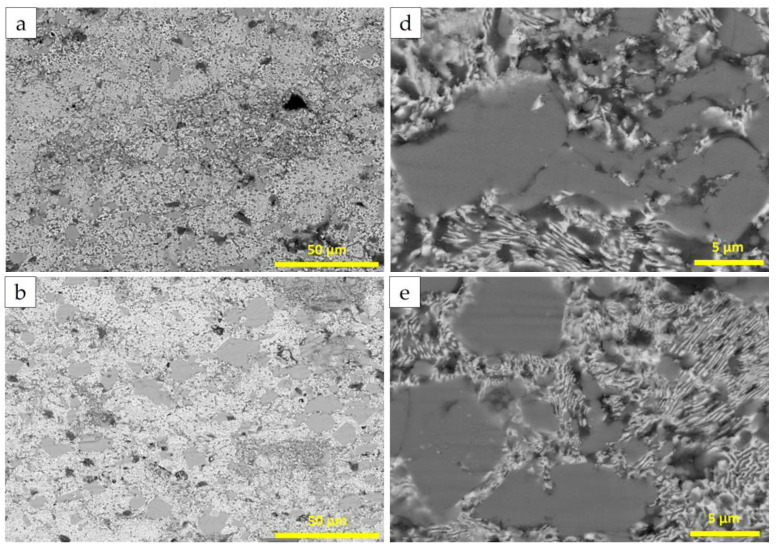

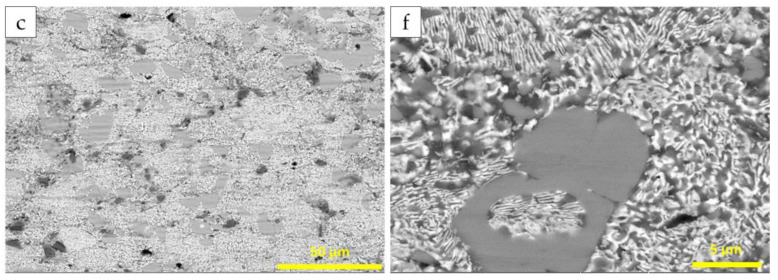
BSE of 20Gr-Ni coating sections with different heat treatment: (**a**,**d**) 200 °C; (**b**,**e**) 300 °C; (**c**,**f**) 400 °C.

Figure 4 shows the microstructure of AlZn/20 nickel-coated graphite coatings after annealing at 200 °C, 300 °C, and 400 °C for 4 h followed by furnace cooling. The annealing process allows diffusion between metal particles, leading to a more uniform coating. The gray areas represent Al particles from the cold-sprayed state, while the bright white areas indicate Zn and Ni accumulation. A significant amount of lamellar structure appears near the Al particles, with even finer lamellar structures forming in the gray phase at 400 °C.

### 3.3. The Effect of Annealing Treatment on Coating Properties

#### 3.3.1. Microhardness

**Figure 5:**
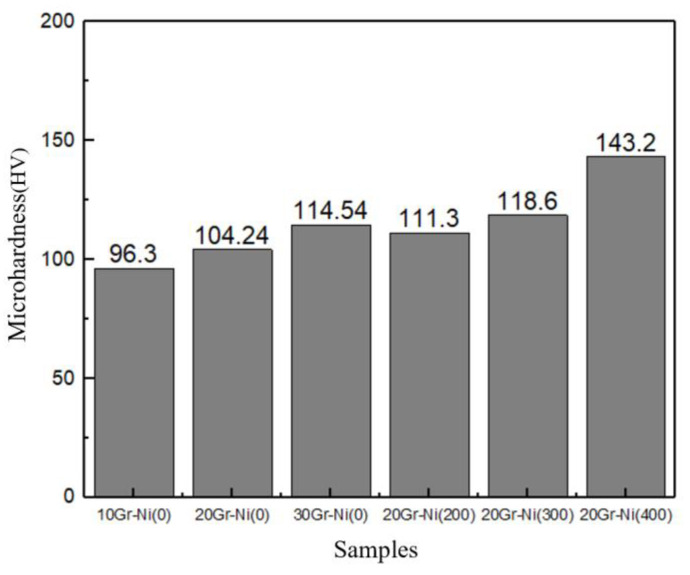
Microhardness of AlZnCu coating.

Figure 5 presents the hardness results for the AlZn/nickel-coated graphite coatings. For the as-sprayed coatings, hardness increased with the addition of nickel-coated graphite, with the highest hardness of 114.54 HV observed in the coating with 30% nickel-coated graphite. This increase is due to the hard nickel-coated graphite particles, which “pin” AlZn particles together and enhance deformation, resulting in work hardening. As deformation increases, the dislocation density within the metal particles rises, leading to dislocation slip and climb as well as grain refinement, which enhances the overall coating strength. After annealing at 200 °C, the hardness slightly increased, with the hardness of the 20% nickel-coated graphite coating rising from 104.24 HV to 111.3 HV. With increasing annealing temperature, hardness gradually increased, reaching 143.2 HV at 400 °C. The figure shows a noticeable lamellar structure near Al particles. Zhang Yangming and others [21] found that AlZn alloys with 5–25% Al (wt.%) are prone to annealing hardening due to the transformation of the Zn-rich α2 phase into an Al-rich α1 phase and a Zn-rich phase, with the latter phases being harder than the α2 phase, leading to an increased coating hardness.

#### 3.3.2. Surface Roughness

Table 5 shows the surface roughness data of AlZn/20 nickel-coated graphite coatings measured by a surface profilometer at different annealing temperatures. The surface roughness of the coating slightly decreases with an increasing annealing temperature. This is primarily due to particles gaining energy during annealing, leading to diffusion. Zn continuously diffuses into Al, strengthening the bonding between particles and resulting in a smoother surface.

#### 3.3.3. Friction Coefficient

**Figure 6:**
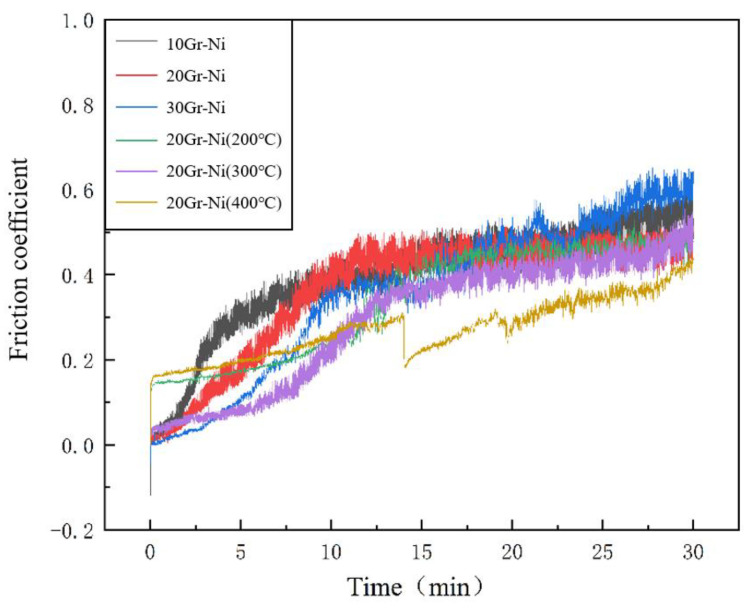
Friction coefficient curves of AlZn/Gr-Ni coatings.

Figure 6 shows the friction coefficient curves for AlZn/nickel-coated graphite coatings. The friction coefficient of the AlZn/nickel-coated graphite coatings remains relatively stable. As observed in Table 6, the average friction coefficient decreases with increasing nickel-coated graphite content. This reduction is attributed to the characteristics of the cold spraying technique, where the high-speed impact of nickel-coated graphite particles accelerates the plastic deformation of Al and Zn particles, leading to a more cohesive coating, as indicated by the earlier porosity measurements. Consequently, during the friction process, particles are less likely to be expelled from the coating, reducing the friction coefficient.

During annealing, metal atom diffusion occurs, transforming the initial mechanical bonding between coating layers into metallurgical bonding, which significantly enhances the bonding strength between layers. Additionally, the reduction in surface roughness makes it harder for the coating to be removed during friction, while the increased hardness at higher annealing temperatures leads to the lowest friction coefficient at 400 °C.

#### 3.3.4. Corrosion Resistance Performance

**Figure 7:**
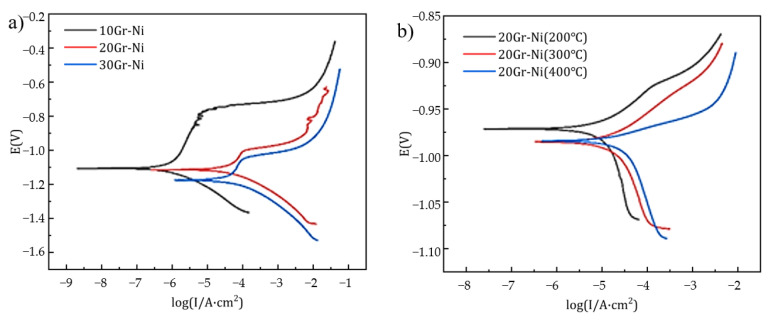
Potentiodynamic polarization curves of AlZn/Gr-Ni coatings in 3.5% NaCl solution: (**a**) different Gr-Ni contents; (**b**) different heat-treatment temperatures.

Figure 7 shows the potentiodynamic polarization curves measured after the open-circuit potentials of the AlZn/nickel-coated graphite coatings stabilized. Table 7 shows the Rp fitting data of AlZn/Gr-Ni. The observation of potential changes reveals that the self-corrosion potential of the annealed coatings generally shifts upward, indicating a lower tendency for corrosion. However, the anodic sections of the polarization curves do not show a distinct passive region, suggesting that no passivation film was formed on the coating surface during corrosion.

This might be due to the initial self-passivation of the coating surface, which raised the self-corrosion potential. Among the coatings, the 10Gr-Ni coating in the cold-sprayed state exhibited the best corrosion resistance. As the nickel-coated graphite content increases, porosity also increases, leading to reduced corrosion resistance. For the 20Gr-Ni coating, although the corrosion potential rises after annealing, the self-corrosion current is slightly higher overall, resulting in a slight decrease in corrosion resistance, but the effect is not significant.

#### 3.3.5. Residual Stress

During the cold spraying process, as powder particles are deposited onto the substrate at high velocities, their kinetic energy is converted into thermal energy and a portion of the deformation work is retained within the metal powder, resulting in residual internal stresses. When the sprayed powder consists of a mixture of different powders, the plastic deformation of the particles can vary, leading to internal stresses between particles. After annealing treatment, the stress state on the coating surface changes. By calculating the residual stress on the coating surface, one can compare the effects of different annealing temperatures on the surface properties of the coating, further optimizing the study of aluminum busbar repair coatings.

In nanoindentation technology, mathematical models are commonly used to calculate the residual stress on a material’s surface. Suresh proposed a theoretical model for measuring residual stress in 1998, focusing on equi-biaxial residual stress [22]. Yun-Hee Lee and colleagues also introduced three models for measuring residual stress in 2002, 2003, and 2004, with the first two models addressing equi-biaxial residual stress and the third extending to non-equi-biaxial, or biaxial stress [23,24,25].

Suresh’s theoretical model assumes that residual stress and residual plastic strain are equi-axial and homogeneous at depths at least several times greater than the indentation size, and that residual stress has no effect on the material’s hardness [22]. The mechanical model is described by the Formula (7):

(7) $$\left(\begin{array}{ccc} \sigma^{R} & 0 & 0\\ 0 & \sigma^{R} & 0\\ 0 & 0 & 0 \end{array}\right)=\left(\begin{array}{ccc} \sigma^{R} & 0 & 0\\ 0 & \sigma^{R} & 0\\ 0 & 0 & \sigma^{R} \end{array}\right)+\left(\begin{array}{ccc} \sigma^{R} & 0 & 0\\ 0 & \sigma^{R} & 0\\ 0 & 0 & -\sigma^{R} \end{array}\right)$$

Due to the presence of two types of residual stress conditions on the material surface—residual tensile stress and residual compressive stress—the formulas for calculating residual stress vary: For residual tensile stress with a fixed load, the calculation formula is given by Equation (8). For residual compressive stress with a fixed indentation depth, the calculation formula is given by Equation (9):

(8) $$\sigma^{R}=H(1-\frac{h_{0}^{2}}{h^{2}})$$

(9) $$\sigma^{R}=H(\frac{A_{0}}{A}-1)$$

where *H* is the material hardness, *h*_0_ is the indentation depth of a sample without residual stress, and *h* is the indentation depth of a sample with residual stress. Correspondingly, *A*_0_ is the indentation area of a sample without residual stress, and *A* is the indentation area of a sample with residual stress.

When residual tensile stress is present:

With a fixed applied load, the calculation formula is given by Equation (10).

With a fixed indentation depth, the calculation formula is given by Equation (11).

(10) $$\sigma^{R}=\frac{H}{\sin\alpha }(\frac{h_{0}^{2}}{h^{2}}-1)$$

(11) $$\sigma^{R}=\frac{H}{\sin\alpha }(1-\frac{A_{0}}{A})$$

**Figure 8:**
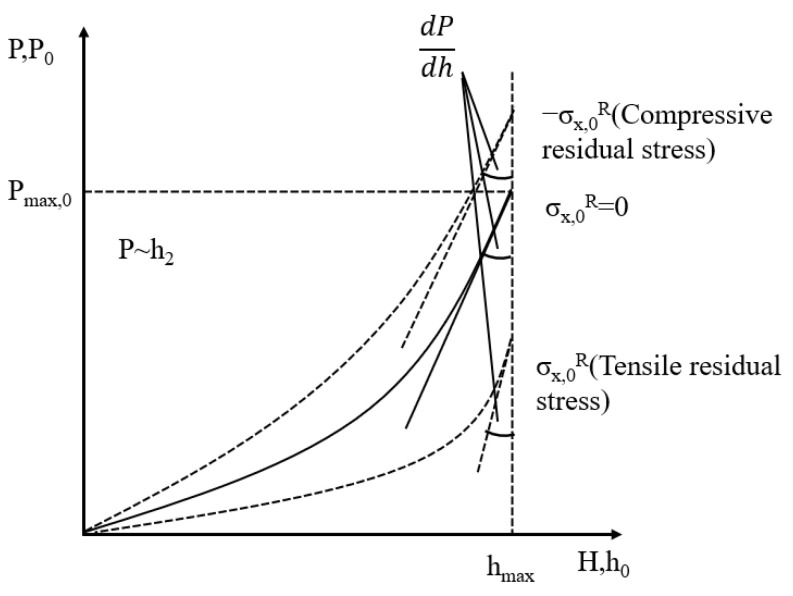
Load–distance curve of material surface without and with residual stress [19].

*α* is the angle between the surface of the indenter and the material surface, varying with different indenter shapes; for example, the Berkovich indenter has an angle of 24.7° with the contact surface. In Suresh’s model [22], a sample without residual stress is needed for reference calculations, typically replaced by an annealed sample in practical computations. To determine the stress state on the material surface, the loading curve from the load–displacement curve of the material surface is analyzed, as shown in Figure 8 [19]:

In Figure 8, the solid line represents the load–displacement curve for a material surface under a no-stress condition. The curves above and below this line represent the load–displacement behavior under compressive and tensile stress conditions, respectively. It is evident that, for a given load, the displacement achieved under compressive stress is less than that under no-stress conditions, which in turn is less than the displacement achieved under tensile stress. Therefore, the relative positions of these curves can be used to determine the stress state on the material surface.

In 2002, Yun-Hee Lee extended the biaxial-stress-state model of Suresh and others by assuming that the hardness remains constant during the experiment, while the slope of the loading curve changes. When the indentation depth is fixed, the appearance of the indentation changes accordingly [23]. This is because equi-biaxial tensile stress can be considered as fluid static stress plus uniaxial stress in the same direction as the indenter load, while equi-biaxial compressive stress can be considered as fluid static stress plus uniaxial stress in the opposite direction of the indenter load. Therefore, as the material surface transitions from tensile stress to no-stress, and then to compressive stress, the indentation surface gradually changes from a depressed state to a piled-up state, and the effective contact area between the indenter and the material also changes. This allows for the calculation of residual stress on the coating surface from the ratio of load difference to effective contact area.

Subsequently, Yun-Hee Lee decomposed the equi-biaxial residual stress tensor into two components: the spherical stress tensor and the deviatoric tensor [24]. The stress model is described by Equation (12):

(12) $$\left(\begin{array}{ccc} \sigma^{R} & 0 & 0\\ 0 & \sigma^{R} & 0\\ 0 & 0 & \sigma^{R} \end{array}\right)=\left(\begin{array}{ccc} \frac{2}{3}\sigma^{R} & 0 & 0\\ 0 & \frac{2}{3}\sigma^{R} & 0\\ 0 & 0 & \frac{2}{3}\sigma^{R} \end{array}\right)+\left(\begin{array}{ccc} \frac{1}{3}\sigma^{R} & 0 & 0\\ 0 & \frac{1}{3}\sigma^{R} & 0\\ 0 & 0 & \frac{1}{3}\sigma^{R} \end{array}\right)$$

In 2004, Yun-Hee Lee expanded on existing research by further subdividing the stress states on the material surface into the following: equi-biaxial stress, non-equi-biaxial stress, uniaxial stress, and pure shear stress. Equi-biaxial and uniaxial stresses are further categorized into tensile and compressive stresses. This led to the development of a new, more comprehensive model for residual stress on material surfaces [25], described by Equation (13):

(13) $$\left(\begin{array}{ccc} \sigma_{x}^{R} & 0 & 0\\ 0 & \sigma_{y}^{R} & 0\\ 0 & 0 & 0 \end{array}\right)=\left(\begin{array}{ccc} \sigma_{x}^{R} & 0 & 0\\ 0 & k\sigma_{x}^{R} & 0\\ 0 & 0 & 0 \end{array}\right)=\left(\begin{array}{ccc} \frac{1+k}{2}\sigma_{x}^{R} & 0 & 0\\ 0 & \frac{1+k}{2}\sigma_{x}^{R} & 0\\ 0 & 0 & 0 \end{array}\right)+\left(\begin{array}{ccc} \frac{1-k}{2}\sigma_{x}^{R} & 0 & 0\\ 0 & \frac{-(1-k)}{2}\sigma_{x}^{R} & 0\\ 0 & 0 & 0 \end{array}\right)$$

**Figure 9:**
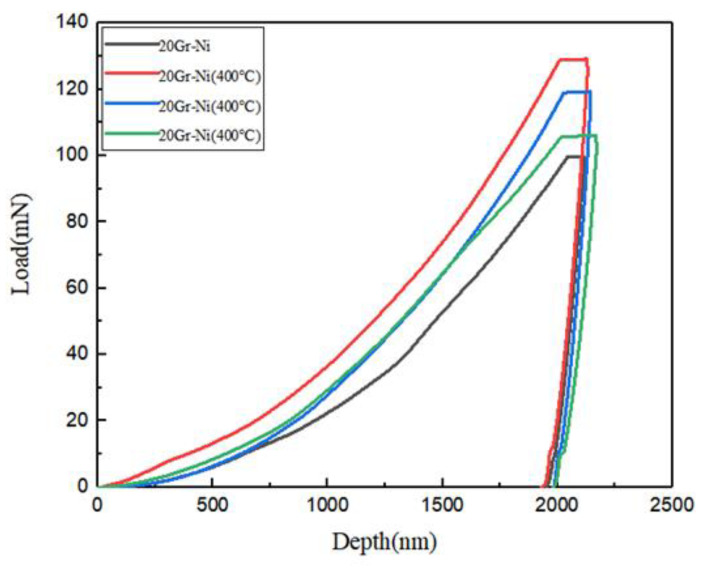
Load distance curves of 20Gr-Ni coatings.

In this experiment, nanoindentation was used to test 20Gr-Ni coatings subjected to different annealing temperatures with a 2000 nm indentation depth. The procedure included the following: (1) loading the indenter at a constant rate of 10 nm/s to the maximum load; (2) maintaining the maximum load for 10 s to minimize the impact of material creep; and (3) unloading at a constant rate of 10 nm/s. Each sample was indented eight times, avoiding defects and areas with uneven composition. The data were fitted after excluding the curves with the maximum and minimum deviations to obtain the load–displacement curves for the cold-sprayed state and annealed coatings at 200 °C, 300 °C, and 400 °C, as shown in Figure 9.

In the experiment, the 20Gr-Ni coating annealed at 200 °C was used as a reference sample with no residual stress, while the cold-sprayed and other temperature-treated samples were tested for residual stress. Table 8 shows the residual stress of Gr-Ni coatings with different heat-treatment temperatures. As the indenter penetrated deeper, the load increased, and a small plateau was observed during the load-holding phase. Upon unloading, the elastic deformation was released, returning to the contact depth. When using the 200 °C annealed sample as the reference, the loading curves of all coatings were below it, indicating that the surface residual stress was compressive, with the maximum residual stress of 0.129 GPa observed at 400 °C. As the temperature increased, the cohesive forces between particles in the coating grew, resulting in higher residual stress values.

## 4. Actual Repair Effect of the Factory

**Figure 10:**
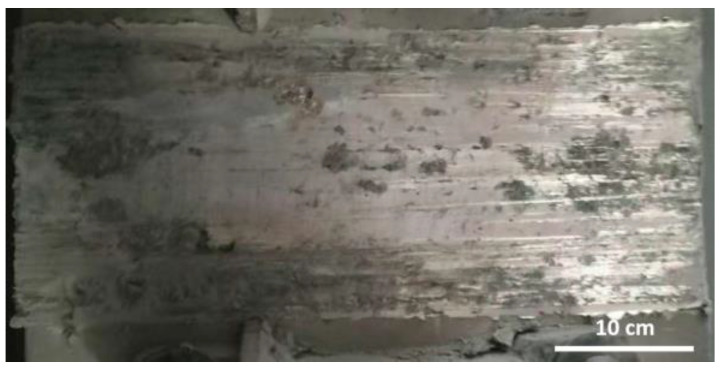
Damaged surface of aluminum bus.

This study is primarily based on the repair work of horizontal busbars at a specific factory. During the practical experimental operations, multiple surface engineering techniques were simulated for repair, but the results were less than ideal. Cold spraying, a novel surface engineering technology, is noted for its convenience and the high density and strong bonding strength of its coatings, offering significant potential in the repair field. Therefore, this study employed cold spraying technology to address the repair needs of horizontal busbars by designing several high-density coatings with good wear resistance and corrosion resistance based on aluminum (Al). Among these, the 30Gr-Ni coating demonstrated the best wear resistance. Ultimately, based on the study results, the AlZn/20Gr-Ni coating, which provides both good wear resistance and adequate corrosion resistance, was selected for field repair. The specific practical operations were as follows:

Pre-treatment of the damaged horizontal busbar surface: Due to the poor surface quality of the damaged horizontal busbar (as shown in Figure 10), the surface of the busbar must be cleaned thoroughly. This is typically performed using a cleaning agent to remove surface oil and contaminants. Alternatively, a grinding machine can be used for mechanical abrasion.

**Figure 11:**
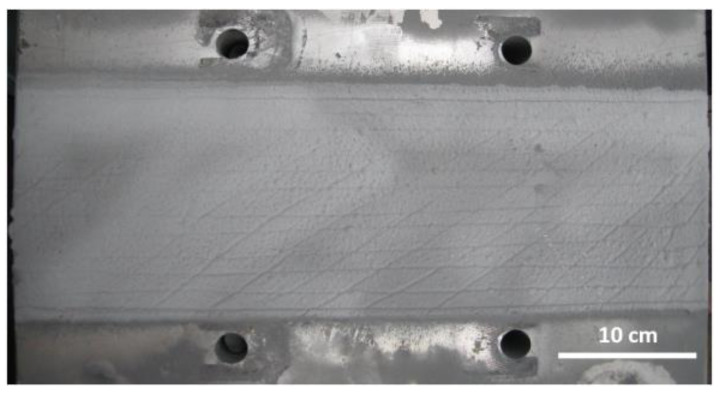
The surface of the aluminum bus after sandblasting.

After polishing, the busbar surface generally requires groove cutting, achieved by filing or using a grinding wheel to create grooves on the polished surface and plane edges. Subsequently, sandblasting is performed to increase the surface roughness before spraying. The sandblasted busbar surface is shown in Figure 11. These processes are intended to ensure that the sprayed powder adheres well to the coating surface and maintains strong adhesion to the substrate.

**Figure 12:**
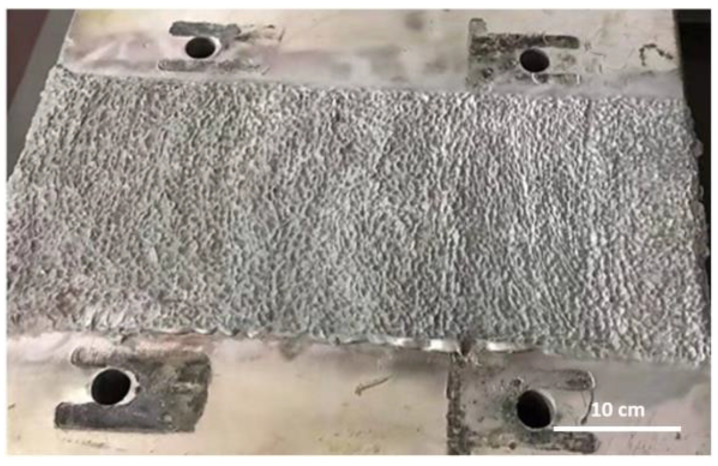
Aluminum bus surface after cold spraying.

Cold spraying: The bus is then treated using low-pressure cold spraying technology, which involves small, portable equipment suitable for on-site spraying. The spraying parameters are the same as those specified in this study. The surface after spraying is shown in Figure 12, where normal particle-impact morphology is observed, with dense bonding and no obvious defects at the edges. The surface requires mechanical processing after spraying before application.

**Figure 13:**
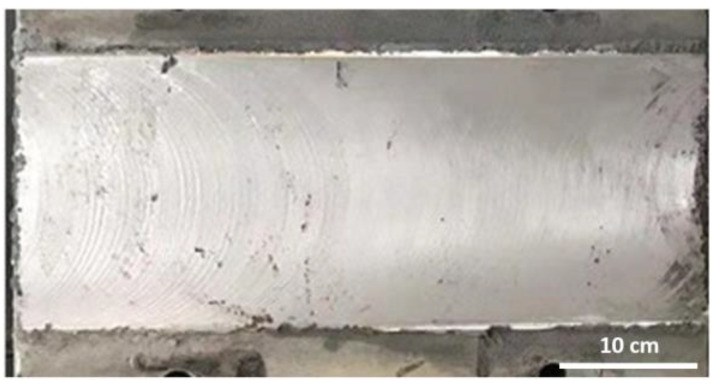
Coating surface after machining.

Coating processing: the coating surface is mechanically machined using milling, and iron oxide red is applied to the surface to check for flatness. The processed coating surface is shown in Figure 13.

**Figure 14:**
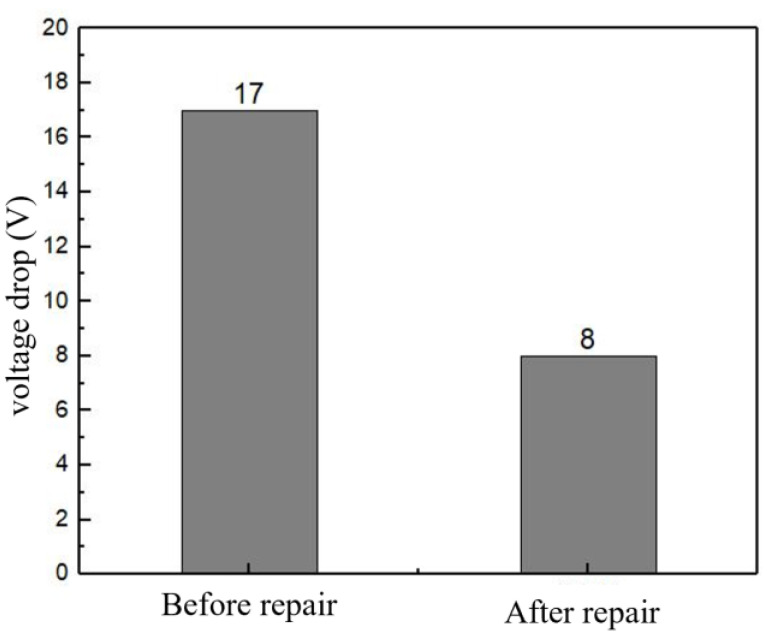
Pressure drop between unrepaired tank and repaired tank.

The coating surface is seen to be smooth and flat, with no micro-pores or cracks, meeting the on-site production requirements. The use of low-pressure cold spraying technology allows for an adjustable coating thickness based on the degree of damage at the site, while the resulting coating surface is dense and free of noticeable defects. After actual field repairs and a period of operational monitoring, a comparison was made between un-grooved and repaired grooves on the busbar. It was observed that the voltage drop between the conductor rod and the busbar decreased from 17 V to 8 V, as shown in Figure 14, effectively reducing energy loss between the conductor rod and the busbar. This indicates that using cold spraying technology to prepare the AlZn/20Gr-Ni aluminum busbar repair layer is successful. This method can be further applied to repair other aluminum busbars and conductor rods within the electrolytic plant, potentially addressing the current issues of low repair efficiency and poor results, and has significant application prospects.

Based on the results from annealing heat-treatment experiments, it is known that annealed coatings have a more uniform distribution and significantly improved wear resistance. Although heat treatment cannot be applied post-repair, this provides a valuable insight for developing auxiliary post-processing techniques for aluminum busbar repair coatings, further meeting the repair needs of busbars.

## 5. Conclusions

This study primarily analyzes the changes in the structure and properties of AlZn composite coatings and explores the effects of different nickel-plated graphite contents on these properties. It also focuses on the comparison of coating structure and performance before and after annealing treatment for 20Gr-Ni coatings at various temperatures. The main conclusions are as follows:

1. Adding nickel-plated graphite to AlZn composite coatings will reduce the porosity with increasing graphite content; the porosities of 10Gr-Ni, 20Gr-Ni and 30Gr-Ni are 2.3%, 2.4%, and 2.8%, respectively.
2. The content of Gr-Ni significantly improves the hardness and friction performance of the coating. Compared with 10Gr-Ni, the hardness of 30Gr-Ni increased by 18.91% and the average friction coefficient decreased by 10.06%.
3. As the content of Gr-Ni increases, the corrosion resistance of the coating decreases. When the content of Gr-Ni increases from 10 wt.% to 30 wt.%, E_0_ decreases from −1.1077 V to −1.1755 V and I_0_ increases from 5.6149 × 10^−7^ A/cm^2^ to 3.5403 × 10^−5^ A/cm^2^.
4. The heat-treatment temperature has a strengthening effect on the hardness and friction performance of the coating. Annealing 20Gr-Ni coatings at different temperatures leads to increased particle diffusion and the formation of layered structures, improving hardness and wear resistance. Compared with 20Gr-Ni, the hardness of 20Gr-Ni (400 °C) increased by 37.38% and the average friction coefficient decreased by 25.92%. The heat-treatment temperature has little effect on the corrosion resistance of the coating. There is no obvious passivation of the coating during the corrosion process.
5. An AlZn/20Gr-Ni coating was used to repair the damaged aluminum busbar. The voltage drop between the conductor rod and the busbar decreased from 17 V to 8 V, demonstrating excellent repair performance.

## Figures and Tables

**Table 1:** Composition and content of 6063 aluminum alloy.

Elements	Fe	Cu	Mg	Mn	Si	Cr	Zn	Al
Content (wt.%)	0.35	0.1	0.45~0.9	0.1	0.2~0.6	0.1	0.1	Bal.

**Table 2:** Composition and content of coating.

Samples	AlZn	Gr-Ni
AlZn/10Gr-Ni	90	10
AlZn/20Gr-Ni	80	20
AlZn/30Gr-Ni	70	30

**Table 3:** Process parameters of cold spraying.

Process Parameters	Values
Preheat Temperature	500 °C
Gas Pressure	0.8 MPa
Spraying Distance	10 mm
Movement Speed	4 mm/s
Gas	Air

**Table 4:** Experimental parameters of nanoindentation.

Parameters	Values
Loading Rate	10 (nm/s)
Indentation Depth	2000 (nm)
Strain Rate	0.05 (L/s)
Frequency	45 (Hz)
Loading Distance	1000 (nm)
Indenter’s Poisson’s Ratio	0.1

**Table 5:** Sa and Sq of 20Gr-Ni coating with different heat-treatment temperatures.

Samples	Sa (μm)	Sq (μm)
20Gr-Ni (0)	13.301	17.087
20Gr-Ni (200 °C)	11.860	14.710
20Gr-Ni (300 °C)	10.735	13.383
20Gr-Ni (400 °C)	10.673	13.278

**Table 6:** Friction coefficients of AlZn/Gr-Ni coatings.

Samples	10Gr-Ni	20Gr-Ni	30Gr-Ni	20Gr-Ni (200 °C)	20Gr-Ni (300 °C)	20Gr-Ni (400 °C)
Average friction coefficient	0.3963	0.3657	0.3564	0.3440	0.2976	0.2709
Maximum friction coefficient	0.6002	0.5161	0.6505	0.5300	0.5540	0.4479

**Table 7:** Rp fitting data of AlZn/Gr-Ni.

Samples	E_0_ (V)	I_0_ (A/cm^2^)
10Gr-Ni	−1.1077	5.6149 × 10^−7^
20Gr-Ni	−1.1152	3.0532 × 10^−5^
30Gr-Ni	−1.1755	3.5403 × 10^−5^
20Gr-Ni (200 °C)	−0.98647	3.3593 × 10^−5^
20Gr-Ni (300 °C)	−0.99527	3.6325 × 10^−5^
20Gr-Ni (400 °C)	−0.98361	5.2916 × 10^−5^

**Table 8:** Residual stress of Gr-Ni coatings with different heat-treatment temperatures.

Samples	P (mN)	H (nm)	A (nm^2^)	$\sigma^{R}$ (GPa)
20Gr-Ni	99.80403	2113.709	9.348 × 10^7^	0.026
20Gr-Ni (200 °C)	129.1211	2128.308	9.121 × 10^7^	0
20Gr-Ni (300 °C)	119.3437	2141.655	9.640 × 10^7^	0.002
20Gr-Ni (400 °C)	106.0804	2166.738	9.598 × 10^7^	0.129

## Data Availability

The original contributions presented in the study are included in the article, further inquiries can be directed to the corresponding authors.
